# Numerical Analysis of Granular Flows in a Silo Bed on Flow Regime Characterization

**DOI:** 10.1371/journal.pone.0119155

**Published:** 2015-03-20

**Authors:** Xingtuan Yang, Nan Gui, Jiyuan Tu, Shengyao Jiang

**Affiliations:** 1 Institute of Nuclear and New Energy Technology of Tsinghua University, and The key laboratory of advanced reactor engineering and safety, Ministry of Education, Beijing, 100084, People’s Republic of China; 2 College of Mechanical and Transportation Engineering, China University of Petroleum, Beijing, People’s Republic of China; 3 School of Aerospace, Mechanical & Manufacturing Engineering, RMIT University, Melbourne, VIC 3083, Australia; Tianjin University, CHINA

## Abstract

The flow characteristics of a gravity-driven dense granular flow in a granular bed with a contracted drainage orifice are studied by using discrete element method and quantitative analysis. Three values of discharging rates, ranging from fast to slow dense flows, are investigated. Time variations and derivatives of mean forces and velocities, as well as their respective correlations, are analyzed to quantitatively depict the characteristics of granular flow as well as flow regime categorization. The auto-correlation functions, as well as their Fourier spectrums, are utilized to characterize the differences between the mechanisms of slow and fast granular flows. Finally, it is suggested that the flow regimes of slow and fast flows can be characterized by the kinetic and kinematic flow properties of particles.

## Introduction

Granular materials behave in a highly complex and unusual manner. They can behave like a solid, a liquid, or a gas. For a solid-like granular media, they can sustain stresses and create a static pile. For a fluid-like system, they can flow like a liquid in an hourglass. Grains can also create a gas when they are strongly agitated [1]. Thus, many different flow regimes can coexist in granular materials. In front of such a complexity, numerous studies have been performed on various aspects of granular assemblies, such as rheology [2], silo quake [3], heterogeneity [4], kinematic shock waves [5], avalanches [6,7], mixing or segregation [8,9], granular friction [10], and relevant measurement techniques [11–13] etc. For example, Gao et al. [11–13] pioneeringly proposed complex network theory to investigate the fluid flows and successfully uncovered the fluid dynamical mechanisms governing the transitions of oil-water/gas-water/oil-gas-water multiphase flow patterns. But the analysis of the fundamental mechanisms in quantitative categorization of granular flow regimes remains to be investigated. Recent comprehensive review on these complex behaviors is provided by Schall & Hecke [14]. Nevertheless, granular materials are still poorly understood because of the complex essences of particle flow and collective phenomena caused by the particle-particle interactions.

The complex aspects as aforementioned are not independent. In reality, they are usually closely correlated to each other. For example, the phenomena and mechanisms related to the intermittency feature of granular flow are, in general, fairly complicated. In the rotating drum problem, Benza et al. [15] showed that hyseresis cycle occurs between an intermittent and a continuous flow regime. They have also discovered a slowing down avalanche duration with a temporal power-law divergence in the intermittent flow regime. Moreover, instead of the hysteretic transition, Fischer et al. [16] observed temporal intermittency in the rotating drum with spontaneous erratic switches from discontinous to continous flow regime.

Such intermittent feature has also been found in other types of flows, such as a collection of rigid frictional disks inside a narrow vertical pipe [17], where the intermittent flow is composed of alternating phases of creep motions when the pressure at the bottom of the granular assembly rises nonlinearly with time and sudden slip. In relation to the specific avalanche characteristic of granular materials, Silbert [4] demonstrated the prevalence of intermittency in gravity-driven, dense granular flows down an inclined plane, etc.

With regards to flow regimes, it can be qualitatively categorized into three principal regimes: a gas-like (rapid flow), a liquid-like (slow flow), and a plastic flow (very slow or quasi-static flow, [14]). These categorizations are always taken to be rather empirical or ideal (asymptotic regime classification based on theoretical dimensional analysis [18]). The analysis of the fundamental mechanisms in quantitative categorization of flow regimes remains to be investigated. Rapid flow has been extensively studied and described by gas-kinetic theories [18,19]. Nevertheless, few insights have been given into the characteristics of slow granular flows as well as their flow regime characterizations.

On the other hand, the pebble-bed high temperature gas-cooled reactor was regarded as the most probable and promising technique for the fourth generation of advanced reactor [20]. The particle flow is fairly slow inside the pebble bed reactor core which belongs to a typical slow flow regime. Thus, more research work is needed to better understand the features of slow particle flow as well as the flow regime characterization. Motived by this consideration, this work aims to provide the underlying complex mechanisms of slow granular flows as well as some important issues on flow regime characterization, via exploring the intermittency characteristics of a slow granular flow comparing to a fast dense flow.

## Methodology

### (1) Discrete element method

The discrete element method (DEM), which is based on the soft-sphere approach proposed by Cundall and Strack [21], is utilized for the present study to numerically simulate the granular dynamics. In general, DEM provides the way to obtain not only the motion trajectories but also the experienced forces of any individual element. With the aid of high performance computer, it becomes a powerful tool to tackle the problems relevant to almost all kinds of particle materials and particle-fluid systems, etc. Thus, it is still under rapid developing over the past decades.

The soft-sphere model accounts for three basic aspects of inter-particle collision mechanisms, i.e. incomplete elastic collision (with a spring coefficient *k* and a restitution coefficient *e*), viscous damping (with a damping coefficient *β*), and friction or sliding trend (with a friction coefficient *γ*). By modelling the contact forces between particles via suitable conservation laws, the motion can be resolved deterministically. The normal and tangential forces can be formulated in accordance with:

$$F_{ji}^{cn}=-k_{n}\cdot \Delta x_{ij}^{n}+\beta_{n}\cdot V_{ji}^{n}$$ (1)

$$F_{ji}^{ct}=-k_{t}\cdot \Delta x_{ij}^{t}+\beta_{t}\cdot V_{ji}^{t}$$ (2)

$${\left|F_{ji}^{ct}\right|}_{\text{max}}\leq \mu \left|F_{ji}^{cn}\right|$$ (3)

where *k* and *β* represent the stiffness and damping coefficient, respectively. *μ* is the friction coefficient. Δ*x*_*ij*_ and *V*_*ji*_ represent the deformation and relative velocity respectively. ‘*n*’ and ‘*t*’ denote normal and tangential components respectively. The parameters are listed in Table 1. Bases on Newton’s law of motion, the linear and angular motion of each particle is tracked subjected to the normal and tangential forces acting on the particle. The validation of the current DEM code has been done in the early work [22–23].

**Table 1:** Parameters used in simulation.

Bed dimensions *B*_*w*_×*B*_*h*_ (*m*)	0.8×1.0
Base angle *θ* (°)	30
Width of outlet orifice *W*_*o*_ (*m*)	120×10^−3^
Particle diameter *d*_*p*_ (*m*)	12×10^−3^
Particle number *N*_*p*_	15210
Friction coefficient *μ*	0.3
Restitution coefficient *e*	0.9
Stiffness factor *K*_*n*_ (*N*·*m* ^−1^)	1×10^4^
Poisson rate	0.3
Time step, *Δt* (*sec*)	1×10^−5^
Total simulated time, *T*_*s*_ (*sec*)	200
Rate of recirculation, *R*_*d*_ (*min* ^−1^)	600; 1200; 6000

### (2) Numerical Setup and simulation conditions

**Fig 1:**
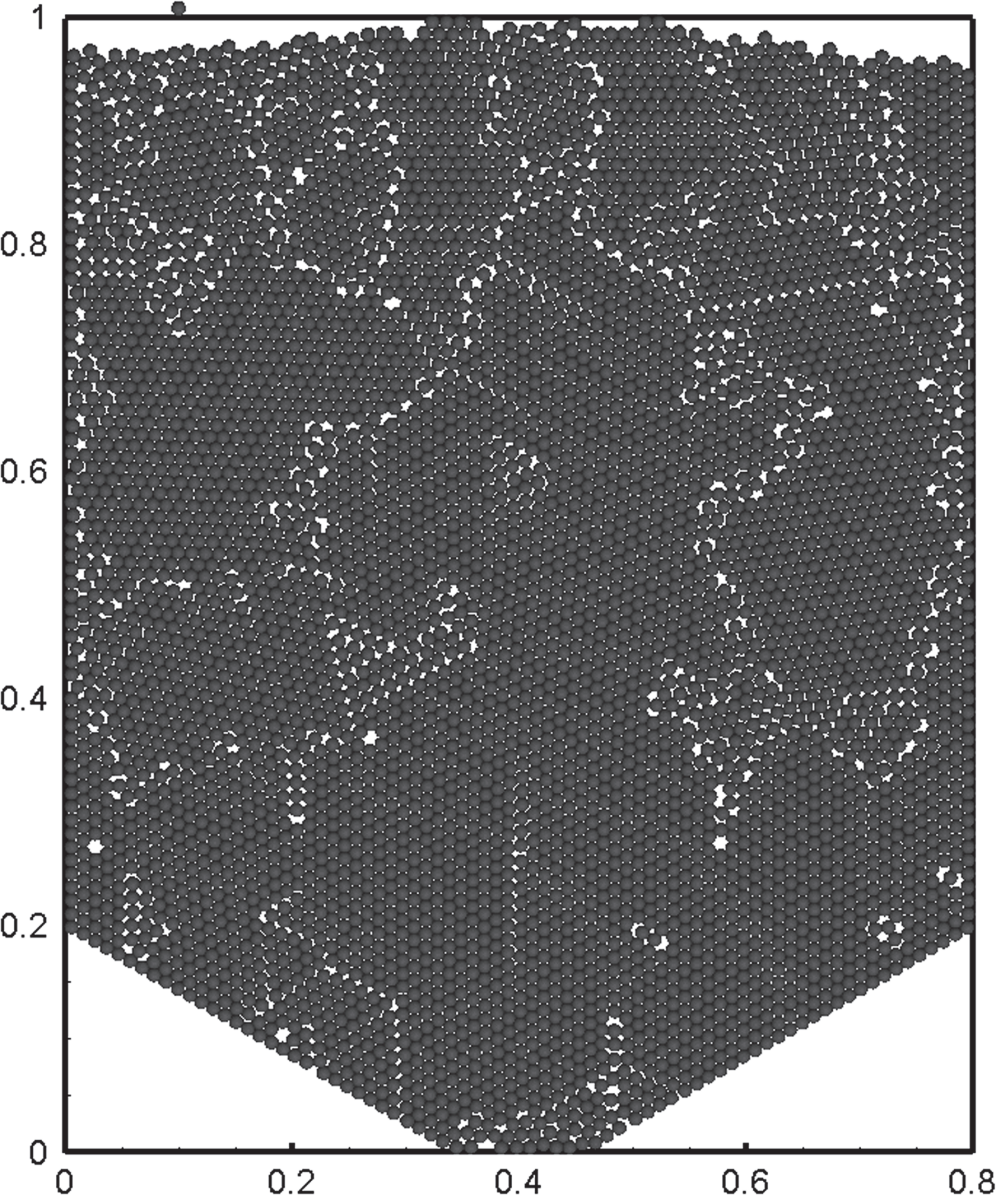
Sketch of numerical setup and demonstration of simulation results.

The dimension of numerical setup is set according to the configurations of a test experimental facility built at INET of Tsinghua university [24], which is 40×800×1000 *mm* in depth (*x*), width (*y*), and height (*z*) respectively. In current simulation, it is filled with 15210 spherical granular particles of diameter *d*_*p*_ = 12 *mm* (Fig. 1). The bottom of the bed is contracted to a drainage orifice with a conical base angle of 30°. A discharging hole of 120 mm in width is located in the bottom centre. The setup runs in a recirculation mode. For each case, the pebbles are removed through the bottom hole at a fixed rate, and simultaneously reloaded at the same rate on the top to keep the number of pebbles stable.

At the beginning, the particles are randomly packed inside the bed. The method for obtaining the randomly packing state can be found in our work [25]. In operation, particles are removed one by one from the bed bottom randomly. After a long time, a stationary recirculation process is established. A statistical analysis is performed to investigate the granular flow behaviour on this so-called time-stationary process.

## Results and Discussions

In this study, three cases with fixed discharging flow rates of *R*_*d*_ = 600, 1200 and 6000 particles/min respectively are investigated for comparative study and analysis. It should be noted that the outflow velocity is averaged at 0.012 *m/s* at the outlet orifice and 0.0018 *m/s* inside the bed for *R*_*d*_ = 600 particles/min since it represents a slow discharging flow. This discharging flow rate is within the parameter ranges which can be achieved by the test experimental facility built at INET of Tsinghua university. For comparative study, a fast flow rate of *R*_*d*_ = 6000 particles/min is used, which is one order of magnitude larger than the slowest one and can be viewed as a relatively rapid dense flow.

In order to probe into the different flow regimes, the essential characteristics of intermittency with regard to granular flows can be correlated through some specific forms of the flow properties. In particular, the numerical data of the contact force and velocity are employed to analyze the flow properties.

### (1) Time evolution of mean force and velocity

The mean contact force and mean velocity of all the particles in the bed can be formulated as:

$$\langle F\rangle =\frac{1}{N_{p}}\sum_{i}\sum_{j}\left|F_{ji}\right|$$ (4)

$$\langle V\rangle =\frac{1}{N_{p}}\sum_{i}\left|V_{i}\right|$$ (5)

where **F**_*ji*_ is the contact force vector from particle ‘*j*’ to particle ‘*i*’, which is dimensionalized by the gravity force magnitude. The operator ‘|•|’ means the norm of a vector, and ‘⟨•⟩’ mean the ensemble averaging procedure.

**Fig 2:**
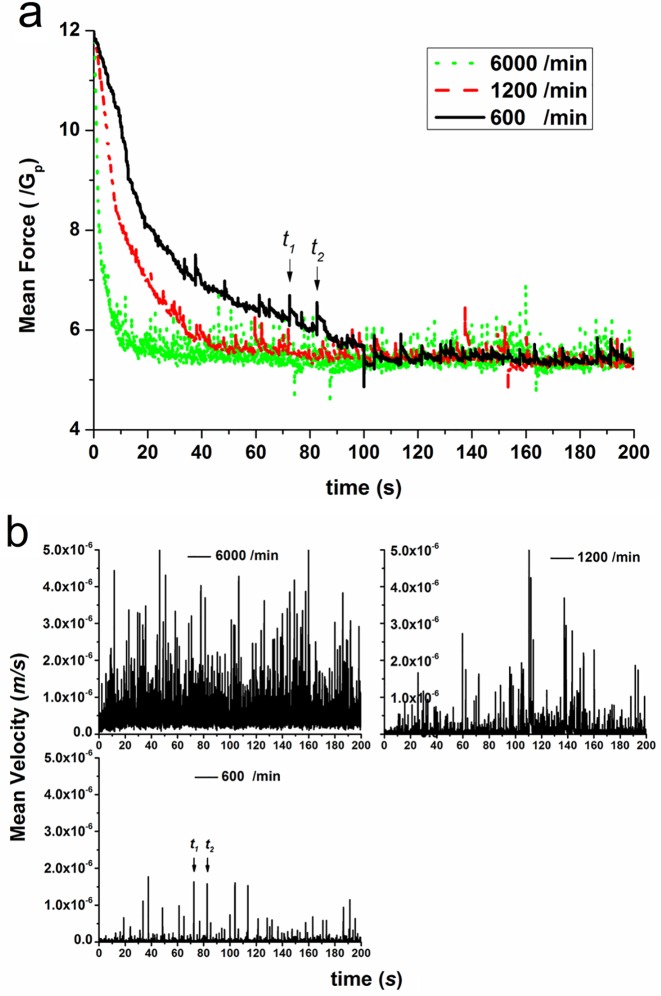
Time variation of mean force (a) and mean velocity (b) of particles.

In Fig. 2A, the time variation of mean contact force demonstrates that:

1. The mean force gradually decreases when the discharging process begins, since the contact force in bed under discharging condition is always smaller than the stationary packing bed. The rate of decrease mainly depends on the discharging rate, i.e. the mean force under the larger discharging rate (*R*_*d*_ = 6000 particles/min) drops faster than the smaller discharging rate (*R*_*d*_ = 600 particles/min);
2. A temporally stationary level of variation of mean contact force is eventually reached, and the stationary levels are the same for different flow rates. This means that the discharging of particles inside the bed has attained a fully-developed condition;
3. More importantly, for all rates, sudden increase of mean force, e.g. pulsing increases of mean force at *t*_1_ and *t*_2_, takes place intermittently for all discharging flow rates, which is always followed by a relatively slowly falling process until a local steady level is reached or an interruption of another impulse occurs.

The time variation of mean velocity for all discharging flow rates is depicted in Fig. 2B. It can be observed that the mean velocities are fluctuating with time. For the high discharging rate (*R*_*d*_ = 6000 particles/min), the fluctuation frequency represents more like a continuous spectrum. In contrast, (*R*_*d*_ = 600 particles/min), large fluctuations are fully separated or spaced out by relatively negligible small fluctuations for low discharging rate.

**Fig 3:**
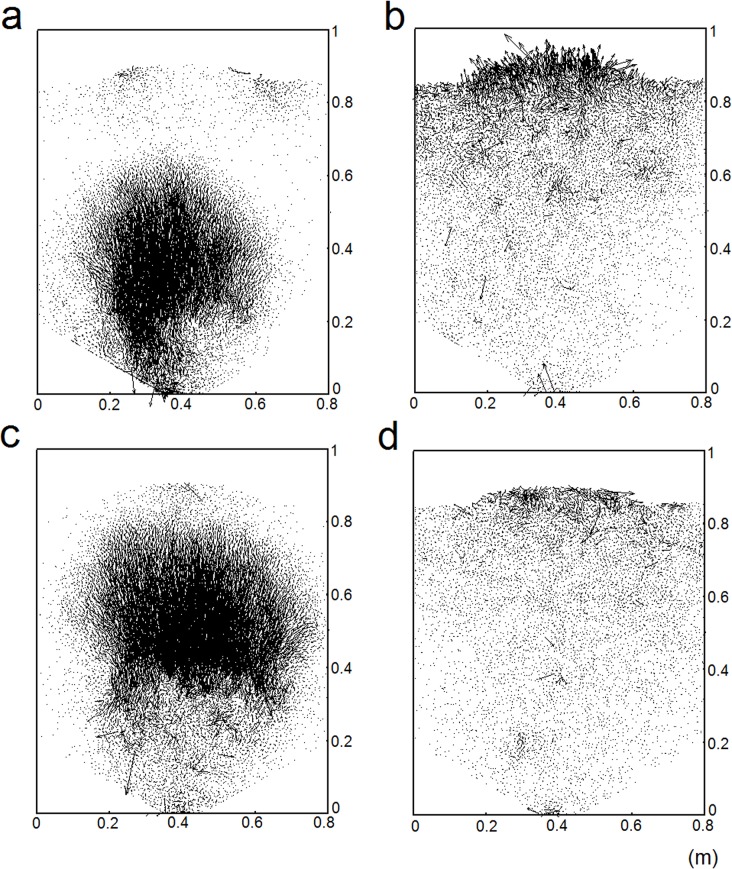
Particle velocity field at *t*_1_ and *t*_2_:_._ (a) *t* = 72.4s; (b) *t* = 72.5s; (c) *t* = 82.6s; (d) *t* = 82.7s.

Some essential characteristics of granular flow may also be obtained via the analysis of the impulse feature of the mean force and velocity. Following this assumption, the states of particles as well as their velocity vectors are shown in Fig. 3A-D, corresponding to the pre- and post-impulse states around *t*_1_ and *t*_2._ Fig. 3A is at *t* = 72.4s (pre-impulse) and Fig. 3B is at *t* = 72.5s (post-impulse) around *t*_1_ = 72.4s. Fig. 3C (pre-impulse) is at *t* = 82.6s and Fig. 3D is at *t* = 82.7s (post-impulse) for *t*_2_ = 82.6s. For pre-impulse states (Fig. 3A and Fig. 3C), large velocity vectors exist inside the bed, which indicate sudden change of large scale distribution structure of particles inside the bed. The mechanisms can be explained in the following:

1. When the particles are being removed at the bottom, the structure of local forces may change slightly and depart from the local force equilibrium. But, its influence is restricted within a local region in the first, because it needs time for the effect of force change to be spread out and in another part because the degree of force structure change is so slight that it does not meet the power threshold to affect the entire global distribution structure of particles. In other words, large scale structure of force or particle distributions is maintained though its strength may be gradually attenuated. The forthcoming change of large scale structure is delayed but the degree of large scale instability is increasing;
2. As time progresses, particles are removed continuously. The force structures and the base at the bottom for supporting the upside particles become weaker. At some time, the sudden change of large scale structure takes place, which behaves like an internal ‘quake’ of particle assembly. This sudden change is always accompanied with an internal bulk motion of falling, during which the overall magnitude of mean velocity of particles must increase.

On the contrary, after the internal ‘bulk motion of falling’, the particles are packed under a relatively tight degree once again inside the bottom region (see Fig. 3B and Fig. 3D), which makes a subsequent sudden increase of the magnitude of mean contact force. This explains why the sudden impulse feature of the mean force and velocity tends to occur at the same time (e.g., pre- and post- *t*_1_ or *t*_2_).

It can be concluded from Fig. 3 that gravity driven granular flow is intrinsically intermittent because the flow and internal structure cannot respond to the instantaneous variation of timely particle discharge. The force structure instability always varies gradually while the large scale structure change of forces usually happens suddenly and intermittently. This means that a delayed response always causes internal sudden changes of essential characteristics of granular flows.

### (2) Time derivative and correlation

In general, a bulk motion of falling is accompanied with a pre-increase of velocity and a post-increase of contact force. Therefore, the variation of mean force and velocity may be correlated to each other. Although there are variations of magnitudes of mean force and velocity (see Fig. 2), the time derivatives may not display much variations and are thus suitable indicators to detect the sudden change of physical parameters, which better represent the change of essential flow characteristics.

**Fig 4:**
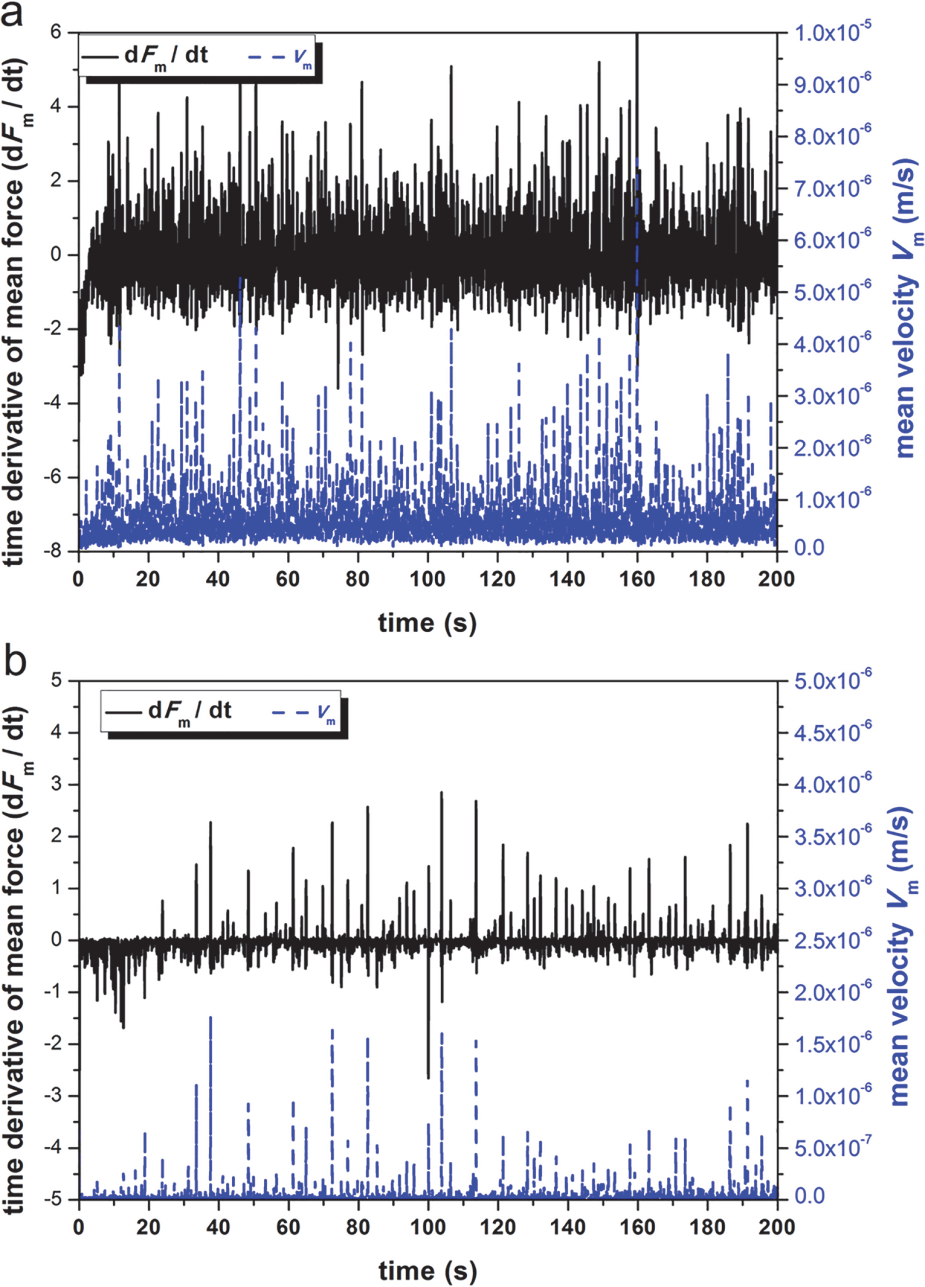
Time derivatives of mean force and velocity for recirculation rate *R*_*d*_ = 6000 /min (a) and 600 /min (b), respectively.

Fig. 4A and 4B illustrate the time derivative of mean force $\left\langle \dot{F}\right\rangle$ and time variation of mean velocity ⟨*V*⟩ for *R*_*d*_ = 6000 and 600 particles/min respectively. The variation of $\left\langle \dot{F}\right\rangle$ is observed to be fairly correlated with the variation of ⟨*V*⟩. The peaks of $\left\langle \dot{F}\right\rangle$ and ⟨*V*⟩ seem to occur statistically at the same time. With the correlation coefficient defined as:

$$\rho \left(\langle \dot{F}\rangle ,\langle V\rangle \right)=\frac{E\left(\langle \dot{F}\rangle -E\left(\langle \dot{F}\rangle \right),\langle V\rangle -E\left(\langle V\rangle \right)\right)}{{\left[D\left(\langle \dot{F}\rangle \right)\cdot D\left(\langle V\rangle \right)\right]}^{\;1/2}}$$ (6)

where ‘*E*(·)’ means expectation, and ‘*D*(·)’ means variance, quantitative values shows that the correlation coefficient ($\rho \left(\langle \dot{F}\rangle ,\langle V\rangle \right)=0.7$) in rapid discharging flows (*R*_*d*_ = 6000 particles/min) is larger than that ($\rho \left(\langle \dot{F}\rangle ,\langle V\rangle \right)<0.5$) in slow flows (*R*_*d*_ = 600 particles/min) (see Table 2).

**Table 2:** Correlation coefficients.

*R*_*d*_ (/min)	$\rho \left(\langle \dot{F}\rangle ,\langle V\rangle \right)$	$\rho \left(\langle \dot{F}\rangle ,\langle V\rangle \right)$
600	0.49	−0.19
1200	0.40	−0.13
6000	0.70	−0.09

The condition of $0.5<\left|\rho \left(\langle \dot{F}\rangle ,\langle V\rangle \right)\right|<0.8$ is considered to be significantly correlated, while the condition of $0.3<\left|\rho \left(\langle \dot{F}\rangle ,\langle V\rangle \right)\right|<0.5$ is considered as lowly correlated. Therefore, it can concluded that $\left\langle \dot{F}\right\rangle$ and ⟨*V*⟩ are significantly correlated for the high discharging flow speed whereas it is lowly correlated for the slow discharging flow speed.

The following analyses provide further explanations of the correlation between $\left\langle \dot{F}\right\rangle$ and ⟨*V*⟩. From Eqs. (1) and (2),

$$\left\langle F\rangle ∼k\langle x\rangle +\eta \langle V\rangle \;\text{and}\;\langle \dot{F}\rangle ∼k\langle \dot{x}\rangle +\eta \langle \dot{V}\rangle ∼k\langle V\rangle +\eta \langle \dot{V}\right\rangle$$ (7)

where ⟨*x*⟩ is the mean deformation of particles. When $\left\langle \dot{F}\right\rangle$ and ⟨*V*⟩ are well correlated, it is appropriate to assume

$$\left\langle \dot{F}\rangle ∼\rho \left(\langle \dot{F}\rangle ,\langle V\rangle \right)\langle V\right\rangle$$ (8)

where $\rho \left(\langle \dot{F}\rangle ,\langle V\rangle \right)$ can be regarded as a constant. Thus, the correlation coefficient $\rho \left(\langle \dot{F}\rangle ,\langle V\rangle \right)$ and stiffness factor *k* are found to play the similar role, provided the $\eta \langle \dot{V}\rangle$ term is negligible. On the other hand, the condition:

$$\eta \langle \dot{V}\rangle \approx 0$$ (9)

indicates either the negligible time derivative of mean velocity or the small relative value of *η* (i.e. *η*/*k* ≈ 3 × 10^−5^ << 1). If mean velocity variation is steady, the velocity derivative is not very large, and $\left\langle \dot{V}\right\rangle /\left\langle V\right\rangle$ would be small. Together with *η*/*k* << 1, it leads to $\eta \langle \dot{V}\rangle /k\langle V\rangle <<1$. Thus, $\left\langle \dot{F}\rangle ∼k\langle V\right\rangle$ and significant correlation between $\left\langle \dot{F}\right\rangle$ and ⟨*V*⟩ will exist.

In addition, the correlation coefficient between $\left\langle \dot{F}\right\rangle$ and $\left\langle \dot{V}\right\rangle$ is found to be fairly small for all flows (see Table 2, $\rho \left(\langle \dot{F}\rangle ,\langle \dot{V}\rangle \right)<0.2$, where *ρ* < 0.3 is usually considered as weakly correlated or even independent). In other words, with $\eta \langle \dot{V}\rangle <<k\langle V\rangle$ and $\left\langle \dot{V}\right\rangle$ independent of $\left\langle \dot{F}\right\rangle$, it guarantees the dominant role of ⟨*V*⟩ in determining $\left\langle \dot{F}\right\rangle$. Thus, they are correlated more or less.

On the other hand, $\rho \left(\langle \dot{F}\rangle ,\langle V\rangle \right)$ is large in rapid granular flow (significant correlation) and small in slow or slow granular flow (low correlation, Table 2). Therefore, the correlation between $\left\langle \dot{F}\right\rangle$ and ⟨*V*⟩ may provide a useful indicator for categorizing the rapid and slow granular flow regimes. When $\left\langle \dot{F}\right\rangle$ and ⟨*V*⟩ are significantly correlated, it is considered as a steady rapid dense flow. Otherwise, it is a slow flow.

### (3) Autocorrelation

To further elucidate the characteristics or mechanisms between the rapid and slow granular flow, the auto-correlation function is introduced to evaluate the time variation of mean force and velocity. As the variables are almost steady in time when *t* >100s (Fig. 2), the auto-correlation function of the stationary time can be obtained as:

$$C_{\varphi \varphi }\left(\tau \right)=\frac{1}{T_{s}}\int_{0}^{T_{s}}\varphi \left(t\right)\varphi \left(t-\tau \right)dt$$ (10)

where *φ* denotes any variable with expectation *E*(*φ*) = 0. The correlation function is normalized by the variance of *φ* to obtain the dimensionless correlation coefficient *ρ_φφ_*. The fast Fourier transformation of *C_φφ_* is the so-called power spectrum:

$$S_{\varphi \varphi }\left(\omega \right)=\int_{0}^{T_{s}}C_{\varphi \varphi }\left(\tau \right)e^{j\omega \tau }d\tau$$ (11)

which illustrates the power distribution characteristics.

**Fig 5:**
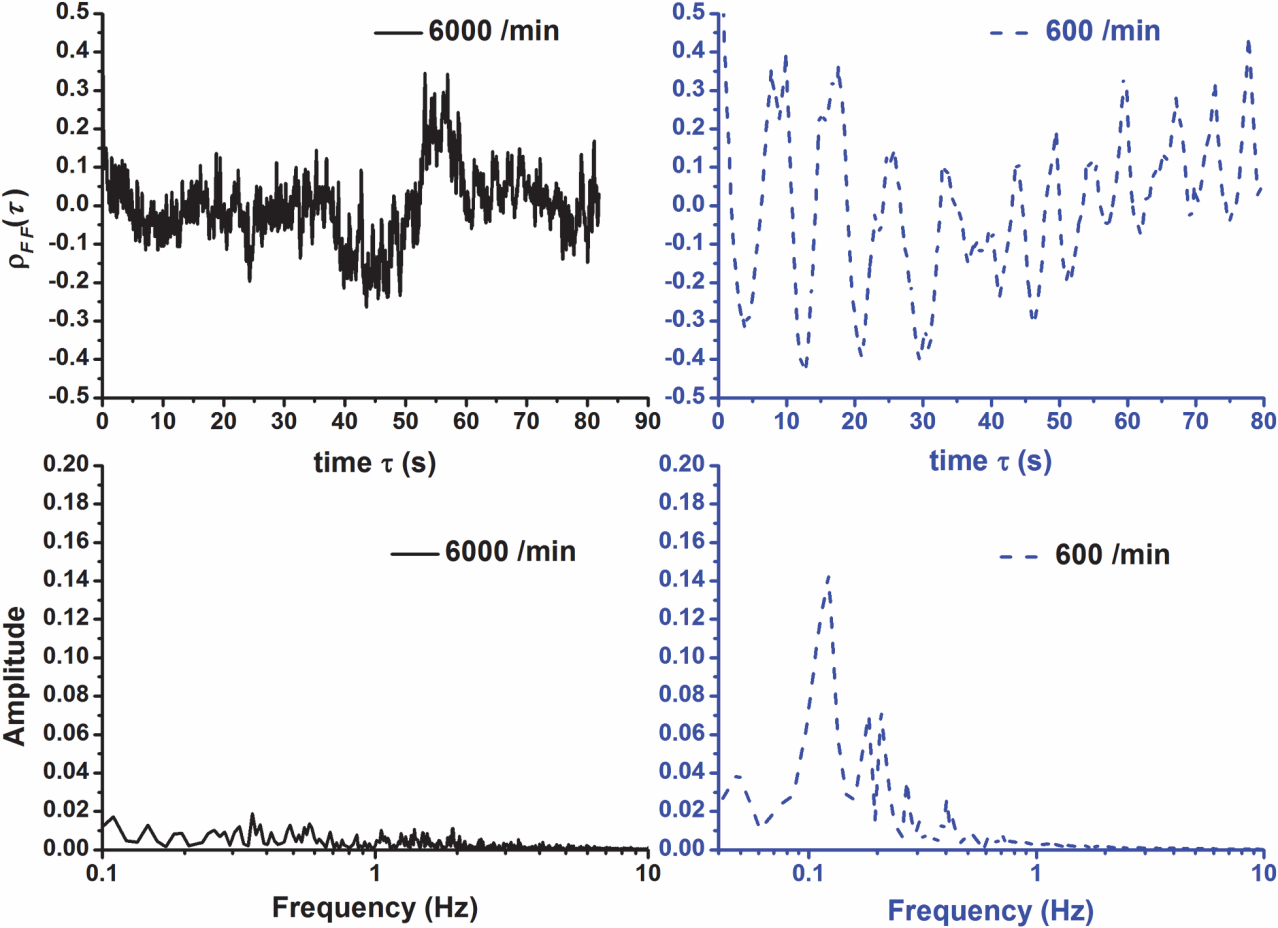
Autocorrelation function and its power spectrum of mean force for *R*_*d*_ = 6000 /min and 600 /min.

**Fig 6:**
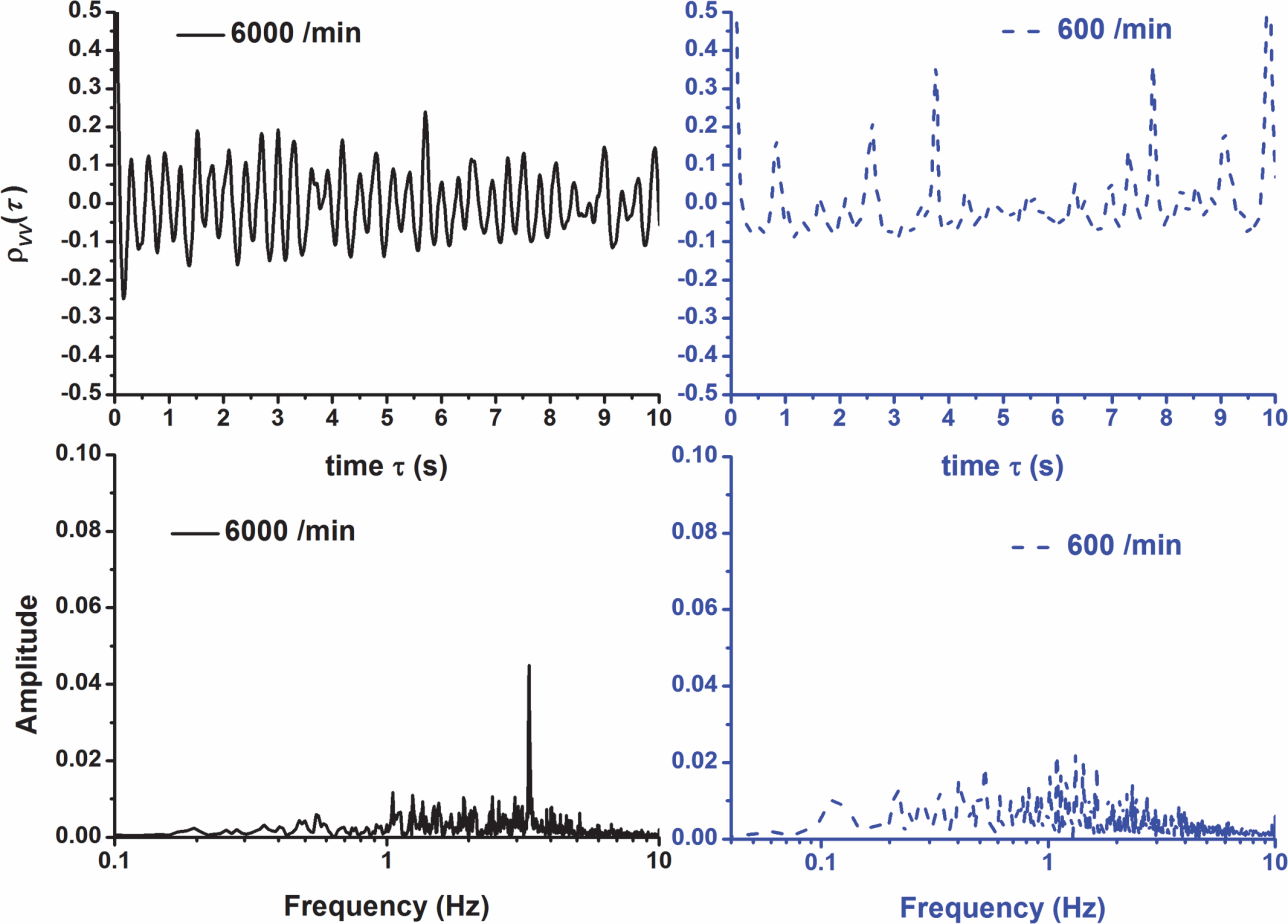
Autocorrelation function and its power spectrum of mean velocity for *R*_*d*_ = 6000 /min and 600 /min.

The autocorrelation function and power spectrum are shown in Figs. 5 and 6 for the mean force and velocities respectively. In Fig. 5, it can be seen that the autocorrelation function of force for *R*_*d*_ = 6000 particles/min fluctuates intensively with time while it only fluctuates moderately and in a more regular fashion for *R*_*d*_ = 600 particles/min. The spectrum shows that the auto-correlation function has an evident fundamental frequency around *f* ^*^ = 0.122Hz with a corresponding characteristic period $T_{f}^{*}=8.2\text{s}$, which is far larger than the period of discharging/recirculating *T*_*d*_ = 0.1s, (with *f*_*d*_ *=* (*R*_*d*_/60s) = 10/s). This means that this characteristic period is not related to the discharging process. Recalling the observation and explanation in Fig. 3, $T_{f}^{*}$ can be explained as the characteristic period of instability, and it is related to the periodic process of the delayed sudden change of large scale force structure. As the particles are removed continuously from the bed bottom and reloaded on the top, the force structure instability is increased in time. After a time period $T_{f}^{*}$, the instability is increased to sufficiently high to cause a sudden change of large structure of force or a sudden internal bulk motion of particles, falling downside. As a result, this time period $T_{f}^{*}$, in average, is regarded as the characteristic period for the particle’s intermittent flow. The absolute value of $T_{f}^{*}$ is related to the discharging rate of particles or the increase rate of instabilities.

Nevertheless, the variation of mean velocity appears to be fair regular for high speed granular flows (*R*_*d*_ = 6000 particles/min), compared to a fairly random variation in low discharging flow speed (*R*_*d*_ = 600 particles/min, Fig. 6). The Fourier spectrum confirms this feature. In high discharging flow speed, the fundamental frequency of variation of mean velocity exists whereas it does not exist in low discharging flow speed. The fundamental frequency and period are *f* ^*^ = 3.333Hz and $T_{f}^{*}=0.3\text{s}$ respectively. In this particular case, the variation of mean velocity is determined mainly by the discharging/recirculating rate. In other words, the constant periodic discharging procedure of particles dominates the variation of mean velocity. The characteristic period for mean velocity variation is 0.3s, and the period of discharging is 0.01 s. Thus, to remove 30 particle in average can cause a sudden change of mean velocity, or cause an internal bulk motion of falling. But this speed of discharging is so fast that the falling particles are discharged from the bottom immediately even before the tightly packed state is recovered. Thus, there is no corresponding periodic variation of mean force and the force is found to fluctuate intensely.

### (4) Further discussion of kinetic regime and kinematic flow regimes

On the basis of the observations and analyses performed, the gravity-driven granular flows can be divided into two regimes:

1. The first regime can be considered as kinetics-characterized regime, termed as slow granular flows. This flow regime is characterized mainly by existence of an evident characteristic period or fundamental frequency of time variation of mean contact force. It is fairly intermittent. The next particle to be removed from the bed in such extended period after the discharge allows the force structures to respond to the change of increasing ‘cavity’ of particle assembly and increasing ‘fault’ or instability of force structures. The large structure of force can maintain its present state until a critical point is met when the force structure subsequently becomes weak. A sudden internal bulk motion of falling and sudden change of force structure occurs. As the contact force is viewed as a dynamic variable, this type of granular flow is known as a kinetics-dominated or kinetics-characterized flow. The intermittency characteristics are mainly related to the dynamic variables.
2. The other regime, which is considered as kinematic flow, is related to fluid-like flow behavior, and termed as fast dense flow regime here. In this type of flow, it is characterized mainly by kinematic variables, such as velocity. The kinematic variables vary rapidly that they exhibit a continuous spectrum. In this particular case, the discharging rate is so high that the force structures are unable to maintain a steady state or form a fully connected stationary packing state before the next particle is discharged. The distribution of particles and force structures could thus always be in an unsteady state. The variation of mean force derivative and velocity are closely correlated. Referring to the fluid-like motion behavior, the correlation of mean force and velocity in fast dense flow is analogous to the correlation of pressure drop (normal internal force) and velocity in fluid flow. Thus, this velocity-dominated or kinematical-variable-characterized flow regime could be described by continuum theories, and the large scale internal flow intermittency is of second importance compared to the slow flow.

## Summary

The categorization of gravity-driven granular flow can be characterized by either kinematic or kinetic flow variables. The kinematic flow regime corresponds to fast dense flow and the kinetic flow is related to slow granular flow.

In the kinematic flow regime, the flow is fluid-like, dynamic stationary, and the flow features are dominated by kinematic variables, e.g. velocities. It can also be characterized by significant correlation between mean force and velocity. However, in the kinetic flow regime, it is a slow flow or quasi-static flow, which is considerably intermittent caused by the internal sudden ‘bulk’ motion of falling accompanied with a sudden change in structures. This implies that the transition from slow to fast regime can be characterized by the transition of existence of characteristic frequency or period of variation of kinetic variable (e.g. contact force) to kinematic variable (e.g. velocity).
